# Patterns of genetic divergence among populations of *Aedes aegypti* L. (Diptera: Culicidae) in the southeastern USA

**DOI:** 10.1186/s13071-019-3769-0

**Published:** 2019-10-30

**Authors:** Kristen A. Hopperstad, Michael H. Reiskind, Paul E. Labadie, Martha O. Burford Reiskind

**Affiliations:** 1 Department of Entomology and Plant Pathology, North Carolina State University, Raleigh, NC USA; 2 Department of Biological Sciences, North Carolina State University, Raleigh, NC USA

**Keywords:** *Aedes aegypti*, Genetic structure, Competitive exclusion, Satyrization

## Abstract

**Background:**

The yellow fever mosquito, *Aedes aegypti* is a public health concern in the USA, especially in the wake of emergent diseases such as Zika and chikungunya. *Aedes aegypti* populations dwindled after the invasion of *Aedes albopictus* in the 1980s and many populations were extirpated. However, in some areas *Ae. aegypti* persisted in small populations and there are reports of recent resurgences of *Ae. aegypti* in Florida, Louisiana, Nevada and California. We assessed the population genetic structure of *Ae. aegypti* in Florida and Georgia, which has concomitant consequences related to mosquito dispersal, pesticide resistance and vectorial capacity.

**Methods:**

We collected *Ae. aegypti* across Florida and in Georgia using ovitraps. We hatched the eggs and reared them to adults, and after sacrifice we extracted their DNA. We then probed each individual for variation in 6 microsatellite markers, which we used to address population genetic characteristics.

**Results:**

We collected *Ae. aegypti* and genotyped seven Florida populations and one Georgia population using microsatellite markers. We found evidence of isolation by distance model of gene flow supported by driving distance among cities within Florida and two theoretic genetic clusters.

**Conclusions:**

Significant genetic structure between some populations with substantial gene flow between geographically distant cities suggests regional genetic structuring of *Ae. aegypti* in Florida. This study provides information on the genetic exchange between populations of *Ae. aegypti* in the southeastern USA and suggests potential routes of spread of this species.

## Background

One of the fundamental questions in ecology is how invasive species affect closely related native species and in the case of mosquito ecology, what the implications are for the spread of disease [1]. Interactions between competitive mosquito species establish the potential for spatial genetic structure and subsequent genetic divergence of populations. The geographical distribution of the naturalized yellow fever mosquito *Aedes aegypti* shifted after the introduction of the Asian tiger mosquito *Aedes albopictus* in 1985 [2]. Although historically *Ae. aegypti* was distributed throughout a large proportion of the southeastern USA in cities, small communities and rural areas [3], it was rapidly replaced throughout most of its range by the spread of *Ae. albopictus* [2, 4]. The rapid extirpation of *Ae. aegypti* was likely due to asymmetric satyrization of *Ae. aegypti* females by *Ae. albopictus* males [5, 6], resulting in interspecific mate competition that favored populations of the invasive *Ae. albopictus* [6, 7]. *Aedes albopictus* also typically outcompetes *Ae. aegypti* as larvae in shared containers, though the outcome of competition is context-dependent [8].

Despite invasion and subsequent extirpation, remnant populations of *Ae. aegypti* remained in areas that ecologically favored *Ae. aegypti*, particularly in urban areas of southern Florida [9–13]. A combination of abiotic factors, including land cover and climate, resulted in areas where desiccation-tolerant *Ae. aegypti* eggs persisted and *Ae. albopictus* eggs could not [14, 15]. In these areas, the outcome of larval competition also shifts to competitive equivalence between the species [16]. Given the history of these interactions between the two species and the potential of detrimental effects to human health, a better understanding of the current population dynamics of *Ae. aegypti* could help inform management strategies.

Areas with high *Ae. aegypti* density, like South Florida, are most at risk of local disease transmission [17]. Recent outbreaks of Zika and chikungunya have re-emphasized the importance of *Ae. aegypti* as a vector for human viruses in the USA, as well as the need for mosquito control to mitigate disease transmission [18, 19]. In addition, *Ae. aegypti* may be spreading out of remnant populations in urban areas of southern Florida, suggesting a reinvasion of its naturalized range [11, 20]. A recent study showed that *Ae. aegypti* is capable of rapidly evolving resistance to interspecific mating with *Ae. albopictus* [7]. Avoidance of interspecific competition and local increases in spatial distributions are cause for concern given that *Ae. aegypti* is a superior vector for many of the viruses that cause human diseases. Therefore, the genetic structure within the southeastern USA, particularly in Florida where *Ae. aegypti* driven outbreaks of human disease have occurred, could help us understand potential routes of reinvasion and disease transmission.

The spatial distribution and genetic structure of *Ae. aegypti* are influenced by its close association with humans [21, 22]. Human-mediated transport allows *Ae. aegypti* to overcome barriers it otherwise could not, as *Ae. aegypti* typically does not disperse much farther than 10–800 m within its lifetime [23, 24]. Urbanization is an important predictor of *Ae. aegypti* habitat suitability and growth, and roads within an urban network play an important role in the rapid spread of *Ae. aegypti* [25]. Roadway systems correspond to patterns of genetic differentiation, with cities linked by major highways being more similar than those not connected in the Bermuda Islands [25], Pakistan [26] and Vietnam [23], and although roads can link disparate populations, they may act as barriers to dispersal at the landscape level [27]. Previous population genetic studies have included southeastern USA populations, though none at a statewide or regional scale [7, 21, 28, 29]. Some studies have not detected significant genetic differentiation between the east and west coasts of Florida or between cities in South Florida (using mtDNA sequences and 12 microsatellite loci, respectively) [21, 29], though a recent study using 5612 variable loci found genetic differentiation among all locations used in their study, even those that were geographically close [7]. Given these contradictory results and evidence of recent reinvasion into its naturalized areas, more comprehensive sampling of *Ae. aegypti*, particularly within Florida, will elucidate population differentiation and possibly identify genetic corridors.

In this study, we use a population genetic approach to clarify the contemporary genetic structure of *Ae. aegypti* in the southeastern USA. Specifically, we focused our sampling on remnant populations of *Ae. aegypti* mosquitoes in Florida and Georgia. Nine highly polymorphic microsatellite loci provided information on the standing genetic structure of this species, how connected isolated populations are to each other and potential mechanisms of dispersal and spread as this species reinvades its naturalized range.

## Methods

### Mosquito collection and rearing

**Fig. 1:**
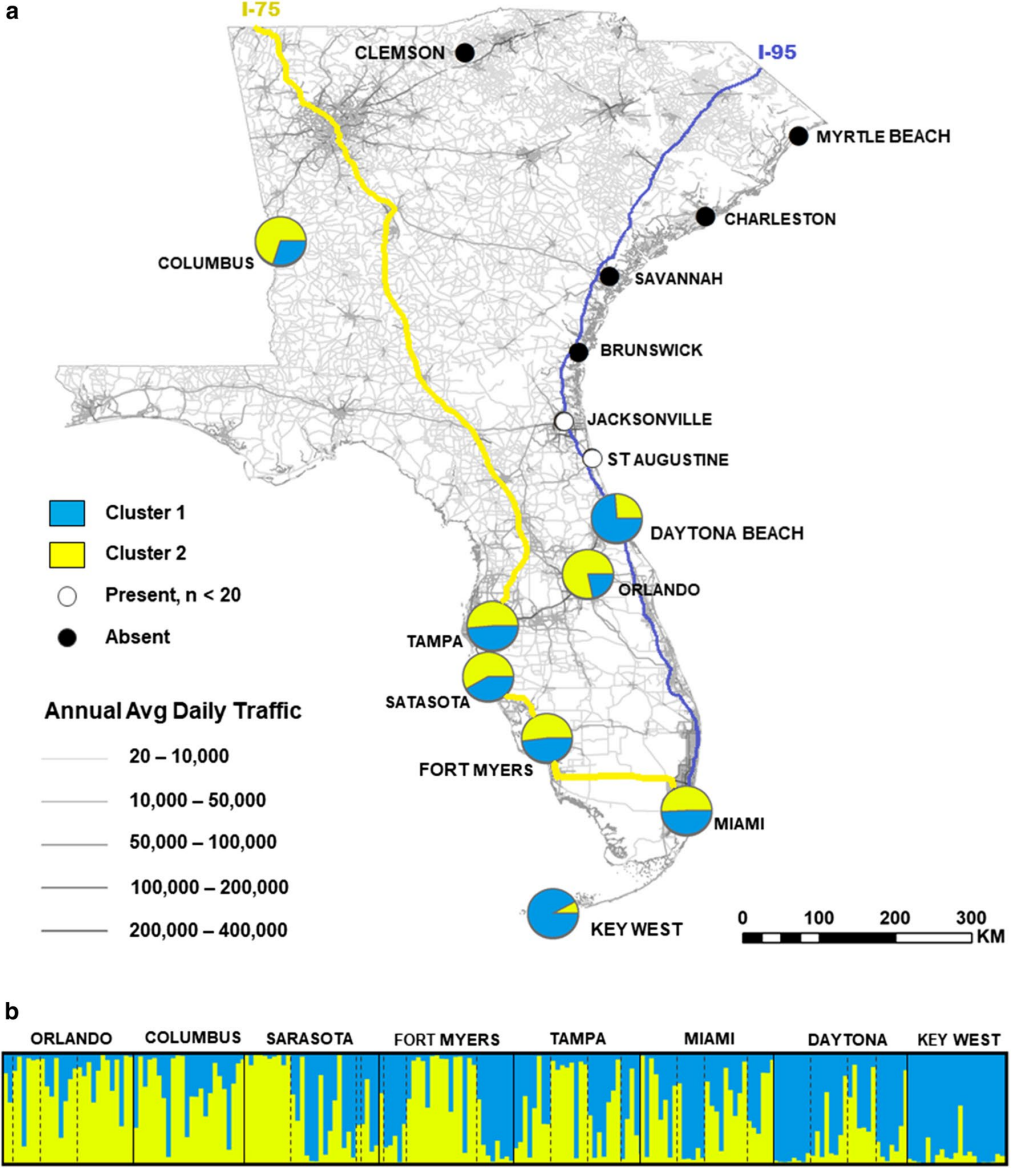
*Aedes aegypti* collection sites and genetic structure using microsatellite loci (*K* = 2). **a** Black circles represent cities in which no *Ae. aegypti* were collected. White circles represent cities in which *Ae. aegypti* were collected, but the sample size was too low to include in the analysis. Pie charts represent a population of *Ae. aegypti* at the city level and the proportion of each color within pie charts corresponds to the mean proportion of ancestry attributable to two theoretic genetic clusters. Mean proportion of ancestry is the average cluster membership of each population for 20 STRUCTURE runs. Grey lines on the map correspond to annual average daily traffic (AADT), symbolized by intensity of line color. The blue line corresponds to Interstate Highway 95 (I-95) and the yellow line corresponds to Interstate Highway 75 (I-75). Traffic and boundary lines are reprinted from Florida Department of Transportation (https://www.fdot.gov/statistics/gis/), the Georgia Department of Transportation (http://www.dot.ga.gov/DS/Data), the South Carolina Department of Transportation (http://info2.scdot.org/sites/GIS) and the National Transportation Research Center at Oak Ridge National Laboratory (https://cta.ornl.gov/transnet/Boundaries.html), accessed 18 Feb 2019. **b** The visual output from STRUCTURE with a *K* = 2. Each vertical bar represents an individual and the proportion of each color represents the proportion of ancestry attributable to two theoretic genetic clusters. Dotted lines separate discrete sampling locations within a city

We surveyed 15 cities in Florida, Georgia and South Carolina for *Ae. aegypti* from June to July in 2014 (Fig. 1a). At each city, we placed 3 to 5 ovitraps in 5 or more locations within urbanized areas of each city. Ovitraps were a minimum distance of 3 m apart and a maximum distance of 140 m apart. Collection locations within cities ranged from 0.25 to 14 km apart (Additional file 1: Figure S1). We collected *Ae. aegypti* eggs and larvae from 9 cities using primarily oviposition cups and supplemented with field collections of larvae and adults. We hatched mosquito eggs at the North Carolina State University Biological Resources Facility insectary and reared mosquitoes to adults for identification and preservation in 95% ethanol at − 20 °C.

### DNA extraction and genotyping

We extracted total nucleic acids from 225 individual *Ae. aegypti* mosquitoes using the DNeasy Blood and Tissue Kit (Qiagen, Hilden, Germany) according to manufacturer instructions, with the exception that we used 30 µl of proteinase K, digested the samples for 72 h and eluted DNA in UltraPure 100 µl DNase/RNase-free distilled water (Thermo Fisher Scientific, Waltham, MA, USA). We quantified template DNA using a fluorometer (Qubit 2.0, Invitrogen, Carlsbad, CA, USA) and tested a suite of polymorphic microsatellite loci. Loci that amplified reliably include four polymorphic loci described by Slotman et al. [30], four polymorphic loci described by Brown et al. [31] and two loci described by Lovin et al. [32] (Additional file 2: Table S1). We paired microsatellite loci based on non-overlapping size ranges and amplified each pair in 15 µl polymerase chain reactions using: 6 µl 2× Promega PCR Master Mix (Promega Corporation, Madison, WI, USA), 1 µl at 10 nM of each fluorescently-labeled M13 forward primer, 1 µl at 10 nM of each reverse primer, 0.4 µl at 10 nM of M13-IRDye (LI-COR Environmental, Lincoln, NE, USA), 3.6 µl of water and 1 µl DNA template. Thermocycler conditions were as follows: 94 °C for 10 min, ×35 cycles (94 °C for 30 s, 54 °C for 30 s, 72 °C for 30 s) and 72 °C for 5 min. We loaded the fluorescently-labeled PCR products onto 6% polyacrylamide gels alongside size standards and detected bands using a LI-COR 4500 automated DNA sequencer (LI-COR Environmental, Lincoln, NE, USA). We hand-scored and hand-binned microsatellite alleles using Gene Profiler v. 4.05 (Scanalytics, Inc., Fairfax, VA, USA) and then screened for scoring errors, allele dropout and presence of null alleles using Micro-checker v. 2.2.3 [33]. Data used in statistical analyses are available in Additional file 3: Text S1.

### Statistical analysis

#### Genetic diversity

We used GENEPOP v. 4.2 [34] to test for deviations from Hardy-Weinberg equilibrium (HWE) genotypic expectations among loci pairs and linkage disequilibrium using exact tests with 10,000 dememorizations, 500 batches and 10,000 iterations per batch. To correct for multiple comparisons, we employed a Bonferroni correction at the 0.05 α level [35]. We calculated observed heterozygosity (*H*_*O*_), expected heterozygosity (*H*_*E*_) and inbreeding coefficient (*F*_*IS*_) using GENEPOP and GenAlex v. 6.5 [36]. We estimated allelic richness by rarefaction for each population using the software HPRARE [37] and calculated the number of private alleles using the R package *poppr v.* 2.8.3 [38].

#### Population structure

We assessed genetic structure among populations using *F*-statistics, including estimates of *F*_*ST*_. We measured genetic differentiation (pairwise exact test, MCMC parameters: 10,000 dememorization, 500 batches, 10,000 iterations per batch) in GENEPOP. We conducted regression analyses of isolation by distance by cluster using linear pairwise $FST\left( {\frac{FST}{1 - FST}} \right)$ values after testing for a normal distribution of the residuals of the regression and pairwise Euclidean and driving distance (km) in JMP.

To identify likely genetic clusters and ancestry, we used a population admixture model in a Bayesian assignment tests implemented by STRUCTURE v.2.3.4 [39, 40]. We conducted 20 independent runs (*n* = 9) for each *K* = 1 to 9 using an initial ‛burn-inʼ period of 40,000 and 100,000 iterations of the MCMC method. We selected the optimal *K* using the *ΔK* method [41] *via* Structure Harvester v. 0.6.94 [42]. We used CLUMPAK online to summarize and graphically represent the STRUCTURE results [43]. We also ran a cluster analysis using a discriminant analysis of principle components (DAPC) using the Adegenet package v. 2.1.1 on RStudio v. 3.5.0 [44]. We used cross-validation with the maximum number of PCs, maximum number of DA axes retained, a 0.1 training set size and 100 repetitions to calculate the optimal number of PCs with the lowest root mean square error (RMSE).

To address a hypothesis about the direction of gene flow between two sample locations connected by Interstate 95 (Dayton and Miami, Fig. 1a), we used the coalescence-based program MIGRATE-N [45, 46]. We tested four migration models: full model with two population sizes of bi-directional gene flow, two population sizes with one-directional gene flow out of Miami, two population sizes with one-directional gene flow into Miami and the null model of panmixia (i.e. the samples came from one population). We used standard parameters and run protocol for microsatellite loci after several trail runs and used a Theta ranging from 0 to 1000, M ranging from 0–1000 and 3,000,000 visited parameter values (a * b * c), using a static heating scheme of 4 chains. Our goal was to address the question of gene flow directionality, not to test for effective population size or absolute number of migrants per generation. We conducted a model comparison using the log Bayes Factor (LBF) based on the accurate marginal likelihoods generated in migrate for the four models [47, 48]. We calculated the marginal likelihoods using the Bezier approximation score generated in MIGRATE-N [47].

## Results

### Mosquito collection data

We sampled 62 sites across 15 cities in the Southeast and recovered *Ae. aegypti* mosquitoes from 30 sites in 10 cities. Although we collected *Ae. aegypti* at sites in Jacksonville and St. Augustine, FL, we did not recover enough individuals to constitute populations and subsequently dropped these cities from the analysis. We selected individuals from multiple ovitraps at four sites per city as evenly as possible. Only one site in Key West and one site in Columbus produced *Ae. aegypti*; however, we were able to supplement Columbus with additional onsite larval and adult sampling. At six sites, only one of the 3–5 ovitraps collected individuals; to address issues of siblinghood in these instances, we restricted the number of individuals included in the analysis from a single ovitrap to a maximum of 10 and average of 5.33 individuals, similar to other studies [31, 49].

### Microsatellite data

After removing individuals with more than 50% missing data, we had 217 individuals (average of 27 per population, Table 1). Due to significant deviation of Hardy-Weinberg expectations (HWE) for most populations, we excluded the 470AG1 locus from all analyses, resulting in nine total microsatellite loci. Micro-checker found no evidence of scoring errors due to stutter, no evidence for large allelic dropout and the presence of few rare null alleles, such that they were negligible. Such findings are common for microsatellites and may explain the low frequency deviations (8.5%) found in violation of HWE [50]. A total of 16 out of 72 population-by-locus comparisons deviated significantly from Hardy-Weinberg expectations (*P* < 0.05, HW exact test) after corrections for multiple comparisons; five showed heterozygote deficiency and one heterozygote excess (Additional file 4: Table S2). However, individual populations deviated from HWE for three or less loci and no loci deviated consistently across all populations.

**Table 1:** Descriptive statistics by population

Population	*n*	*H*_*E*_	*H*_*O*_	*F*_*IS*_	AR	PA
Columbus	24	0.581 ± 0.036	0.515 ± 0.072	0.136	3.85	0
Daytona	29	0.584 ± 0.045	0.516 ± 0.058	0.132	4.06	0
Orlando	28	0.608 ± 0.056	0.602 ± 0.047	0.034	4.30	4
Tampa	28	0.627 ± 0.046	0.514 ± 0.048	0.202	4.53	1
Sarasota	28	0.647 ± 0.031	0.601 ± 0.069	0.092	4.27	0
Fort Myers	28	0.630 ± 0.041	0.595 ± 0.068	0.080	4.21	1
Miami	29	0.613 ± 0.035	0.582 ± 0.037	0.070	4.06	0
Key West	21	0.609 ± 0.052	0.688 ± 0.072	− 0.110	4.02	0
Overall	26.88	0.613 ± 0.015	0.577 ± 0.021	0.080	4.16	6

*Abbreviations*: n, no. of individuals per population; *H*_*E*_, expected genetic diversity; *H*_*O*_, observed heterozygosity; *F*_*IS*_, inbreeding coefficient; AR, allelic richness estimated by rarefaction (*n* = 21 genes); PA, no. of private alleles

### Genetic diversity

We assessed genetic diversity by estimating heterozygosity values and allelic richness by populations and loci (Tables 1 and 2). Observed heterozygosity (*H*_*O*_) and expected heterozygosity (*H*_*E*_) were not significantly different (Student’s t-test, *P* = 0.183; Table 2). Key West showed the only negative *F*_*IS*_ value, indicating less relatedness than expected under a model of random mating. We also found over two times higher *F*_*IS*_ at Tampa than any other location indicating higher effects of genetic drift than other locations. We did not find differences in allelic richness estimated by rarefaction (*n* = 21) among the eight populations (Table 1).

**Table 2:** Descriptive statistics by locus

Locus	*n*	*H*_*E*_	*H*_*O*_	*F*_*IS*_	AR
A1	217	0.651 ± 0.019	0.577 ± 0.049	0.115	5
AC1	216	0.663 ± 0.023	0.669 ± 0.061	− 0.009	5
AC2	216	0.452 ± 0.044	0.465 ± 0.051	− 0.027	4
AC5	215	0.769 ± 0.013	0.668 ± 0.038	0.132	9
A9	211	0.569 ± 0.036	0.453 ± 0.044	0.203	4
B2	195	0.668 ± 0.027	0.811 ± 0.039	− 0.214	8
B3	206	0.602 ± 0.020	0.548 ± 0.059	0.089	4
CT2	204	0.461 ± 0.019	0.403 ± 0.049	0.126	3
1132CT1	207	0.678 ± 0.044	0.596 ± 0.057	0.121	17
Overall	209.67	0.613 ± 0.015	0.577 ± 0.021	0.060	6.56

*Abbreviations*: n, total no. of loci that amplified for all individuals; *H*_*E*_, expected genetic diversity; *H*_*O*_, observed heterozygosity; *F*_*IS*_, inbreeding coefficient; AR, the total no. of alleles per locus

### Genetic differentiation and structure

Results of the exact test in GENEPOP with corrections for multiple comparisons showed significant pairwise differences for all populations except Daytona Beach and Miami (*F*_*ST*_ = 0.010), despite a geographical distance of roughly 640 km (Table 3). Key West was the most differentiated from other locations, followed by Columbus (Table 3). We found a significant signature of isolation by distance for the Florida samples for both Euclidean distance (*F*_(1, 27)_ = 13.43, *P* = 0.001) and driving distance (*F*_(1, 27)_ = 23.19, *P* < 0.001), although for Euclidean distance the residuals violated the assumption of a normal distribution. This violation was driven by a single pairwise comparison between Columbus and Key West (Additional file 5: Figure S2).

**Table 3:** Pairwise *F*_*ST*_ for study locations

	Columbus	Daytona	Orlando	Tampa	Sarasota	Fort Myers	Miami
Daytona	0.066*						
Orlando	0.052*	0.063*					
Tampa	0.075*	0.040*	0.033*				
Sarasota	0.044*	0.043*	0.032*	0.040*			
Fort Myers	0.077*	0.044*	0.034*	0.023*	0.029*		
Miami	0.052*	**0.010**	0.069*	0.045*	0.039*	0.034*	
Key West	0.156*	0.066*	0.105*	0.066*	0.094*	0.052*	0.073*

*P < 0.0001 according to a pairwise exact test. The only non-significant pair (Miami and Daytona Beach) is in boldface

STRUCTURE Harvester determined two distinct theoretical genetic clusters (Net nucleotide distance = 0.044, *ΔK* = 2; Fig 1b; Additional file 6: Figure S3). Daytona, Miami and Key West group into one cluster and the remaining populations group into a second cluster, with a considerable amount of variation. There was appreciable variation within cities; the STRUCTURE plot in Fig. 1b shows sampling sites within cities separated by dashed lines. The DAPC cluster analysis using 40 PCs (mean success = 0.509, RMSE = 0.502) showed similar results, with substantial overlap for Florida populations and Key West again the most differentiated population (Additional file 7: Figure S4).

We found the highest probability (99%) for the one-way directional model of gene flow out of Miami to Daytona. The log Bayes Factor (LBF) was highest for the out-of-Miami model at 34,210 and next for the into-Miami model at 4561. These results supported the hypothesis of gene flow from the major urban center of Miami to Daytona (following the driving distance along Interstate Highway 95) as opposed to the other three models of gene flow into Miami, bi-directional gene flow or panmixia.

## Discussion

*Aedes aegypti* showed significant genetic structuring among all populations, with the exception of the comparison between Daytona Beach and Miami. In addition, we found evidence of the isolation-by-distance model of gene flow supported by driving distance among cities within Florida and two theoretical genetic clusters. The genetic clusters of the two populations within the Southeast appear to visually correspond to major roadways in eastern Florida (Interstate 95 Highway, Highway 100) and western Florida (Interstate 75 Highway; Fig 1a). The observed patterns may also be due to differentiation across space, which could explain in part the low *F*_*ST*_ values [51]. While previous studies found little to no genetic structure of *Ae. aegypti* within Florida, here we show significant genetic differentiation among populations within the Southeast. This further extends the results of the geographically limited study of Burford Reiskind et al. [7], which also found significant genetic structure among four populations within Florida.

### Long-distance connectivity

We detected significant genetic differentiation among populations except between Miami and Daytona Beach, suggesting higher levels of gene flow between these two cities. Roughly 640 km separate Miami and Daytona Beach, but Interstate 95 Highway links the two cities. Considering the nature of this mosquito as a long-distance disperser *via* human transportation, it is possible that vehicular traffic between Miami and Daytona Beach contributes to connectivity between these disparate populations. Here we show that the directionality of this movement was out of Miami towards Daytona. If we classify urban centers as islands, this result shows an expected pattern of large to small island movement of mosquitoes. Considering the larger populations of humans and density of *Ae. aegypti* in Miami, this would be expected. Human-mediated transport allows *Ae. aegypti* to overcome barriers it otherwise could not [23, 26], and roadway systems correspond to patterns of genetic differentiation, with cities linked by major highways being more similar than those not connected [25, 52]. Connectivity between Miami and Daytona Beach was not surprising as gene flow between populations of *Ae. aegypti* can be maintained over remarkably broad scales. Goncalves da Silva et al. [53] found a direct connection between North America and southeastern Brazil, likely *via* sea trade. At a narrower scale within Brazil, they detected extensive gene flow among major cities, with the city of Manaus serving as an important hub connecting a regional network [53]. Here, we found an overwhelming pattern of isolation by distance, with human movement patterns explaining long-distance transport between two major cities.

### Fine-scale population differentiation

While we found lower observed heterozygosity in many of our populations, which could be explained by local inbreeding at locations such as Tampa, a pattern of within-population genetic structure could also cause this pattern [54]. Considering the highly focal nature of *Ae. aegypti* [24] and relatively low effective population size [55], there may genetic differentiation at a finer scale, such as within cities. The STRUCTURE analysis for Florida showed individuals grouped in order of collection site within cities (Fig. 1b, individual collection sites separated by dashed lines). There are apparent differences between sampling sites within cities, particularly in Sarasota and Fort Myers, although given the lower numbers of individuals per sampling site within cities we lack statistical power to detect significant differences. This pattern may also explain the lower relatedness among individuals at Key West, although more thorough sampling would help elucidate whether this was a consistent pattern. Other studies have investigated fine-scale differentiation, such as Hemme et al. [27], which detected significant population structure on either side of a highway. Burford Reiskind et al. [7] found mosquito populations in Apopka (FL) and Kissimmee (FL) were genetically differentiated despite being only ~ 55 km apart [7]. However, Brown et al. [29] measured the phylogeography and spatio-temporal genetic variation of *Ae. aegypti* along Highway 100 from Key West to Miami and found high genetic similarity among all populations [29]. This is likely explained by high levels of tourist and commercial traffic between Miami and Key West. To properly assess within-city structure for cities in this study, additional sampling and explicit hypothesis testing of within-city divergence are necessary.

### Future research

Without a historical baseline of population genetic structure prior to *Ae. albopictus* invasion, it is difficult to assess whether *Ae. albopictus* influenced the population differentiation of *Ae. aegypti*. *Aedes aegypti* was not ubiquitous across Florida pre-*Ae. albopictus* invasion [3] and historical differentiation patterns are unknown. High mobility, rapid generation times and genetic temporal instability in areas of high traffic [55] coupled with the high cost of mating interference avoidance behavior [56], renders satyrization an unlikely source of widespread population differentiation. However, interactions at a local level do have genetic consequences [7, 56]. The incorporation of pre-invasion specimens, such as from museums, could untangle the genetic consequences of *Ae. albopictus* invasion at the population level, as may comparing populations that have and have not been exposed to *Ae. albopictus*.

Sampling additional populations in the Southeast and the inclusion of more sampling sites per population would further resolve structure and cluster membership. Moreover, including a landscape/cityscape genetic approach would help determine corridors of connectivity among populations. In this way, we can better understand how populations in the Southeast remain differentiated despite rapid generation times and long-distance dispersal *via* human transportation. More intensive within-city sampling would reveal whether there are true differences between sampling sites within cities and explicit hypothesis testing of landscape-level barriers to gene flow may reveal potential mechanisms. The incorporation of GIS and traffic data could evaluate the importance of human transportation on Florida population structure. Further, linking phenotypic data, such as pesticide resistance as determined by CDC bottle bioassay, could help us understand patterns of resistance as they relate to genetic connectivity.

## Conclusions

Our results show significant genetic structure between all populations and substantial gene flow between geographically distant cities, which suggests genetic structuring of *Ae. aegypti* at a regional scale. This study serves as a baseline for understanding the structure of Florida populations and can *a priori* inform questions related to landscape influence on interconnectivity. This study and others like it add to the knowledge base regarding *Ae. aegypti* genetic structure, which has concomitant consequences related to mosquito dispersal, pesticide resistance and vectorial capacity.

## Supplementary information

**Additional file 1: Figure S1.** *Aedes aegypti* collection locations within cities. Multiple ovitraps and/or larval/adult sampling were conducted at each collection location. Black circles indicate *Ae. aegypti* specimens were collected at that location and were used in this study. White circles indicate no *Ae. aegypti* specimens were recovered from that location. Points are overlaid 2017 NAIP imagery, reprinted from the USGS (https://catalog.data.gov/dataset/usgs-naipplus-overlay-map-service-from-the-national-map), public domain, original copyright 2017.

**Additional file 2: Table S1.** Microsatellite loci used in study. Loci pairs are based on non-overlapping size ranges. Size range is derived from a literature review [30–32, 55]. 470AG1 was excluded due to significant deviations from Hardy-Weinberg.

**Additional file 3: Text S1.** Microsatellite data used in analyses in Genpop format. Microsatellite data are from all populations indicated in Table 1. Missing data are indicated with 0000.

**Additional file 4: Table S2.** Population-by-locus genetic diversity of microsatellite markers in eight *Aedes aegypti* populations. *N* is the number of individuals, *N*_*A*_ is number of alleles, *H*_*O*_ is observed heterozygosity, *H*_*E*_ is expected heterozygosity, and *P* is *P*-value of an exact test. Note that * indicates *P* < 0.05 with corrections for multiple comparisons; *d* is heterozygote deficiency and *e* is heterozygote excess.

**Additional file 5: Figure S2.** Signature of isolation by distance for both Euclidean distance and driving distance. Scatterplots of Euclidean distance (**a**) and driving distances (**b**) by pairwise linear *F*_*ST*_ with fitted linear regressions superimposed.

**Additional file 6: Figure S3.** Delta K analysis of the true number of clusters following the Evanno method.

**Additional file 7: Figure S4.** Discriminant analysis of principal components (DAPC). Analysis with 40 principal components and a cross-validation of 100 iterations (mean success = 0.509, RMSE = 0.502).

## Data Availability

Data supporting the conclusions of this article are included within the article and its additional files. The dataset studied is included in Additional file 3.

## Abbreviations

AR: allelic richness
PA: private alleles
DAPC: discriminant analysis of principal components
F_IS_: inbreeding coefficient
F_ST_: fixation index
H_E_: expected heterozygosity
H_O_: observed heterozygosity
N: no. of individuals
NA: no. of alleles
RMSE: root mean square error
LBF: log Bayes factor, $2[{ \ln }\left( {mL\left( {model1} \right) - \ln \left( {mL\left( {model2} \right)} \right)} \right]$
