# Supplementary material for: Patterns of genetic divergence among populations of Aedes aegypti L. (Diptera: Culicidae) in the southeastern USA
Source: Parasit Vectors. 2019 Oct 30;12:511. doi: 10.1186/s13071-019-3769-0 (PMC6822358; doi:10.1186/s13071-019-3769-0)
Supplement: Supplementary file 1 — Additional file 1: Figure S1. Aedes aegypti collection locations within cities. Multiple ovitraps and/or larval/adult sampling were conducted at each collection location. Black circles indicate Ae. aegypti specimens were collected at that location and were used in this study. White circles indicate no Ae. aegypti specimens were recovered from that location. Points are overlaid 2017 NAIP imagery, reprinted from the USGS (https://catalog.data.gov/dataset/usgs-naipplus-overlay-map-service-from-the-national-map), public domain, original copyright 2017. [file 13071_2019_3769_MOESM1_ESM.pdf]

Columbus, GA

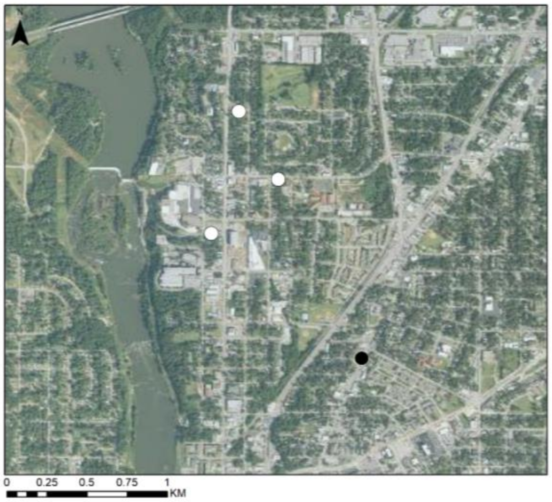

Daytona Beach, FL

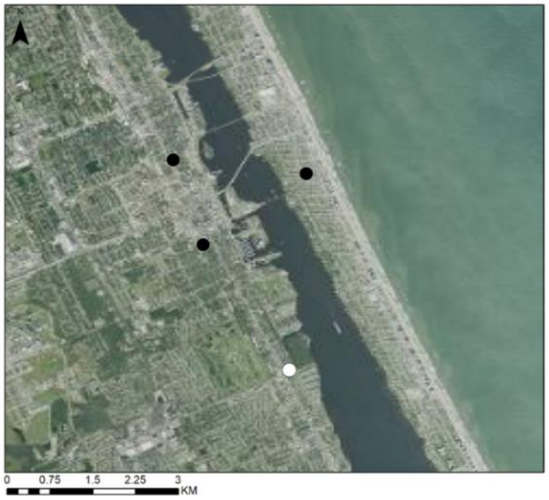

Orlando, FL

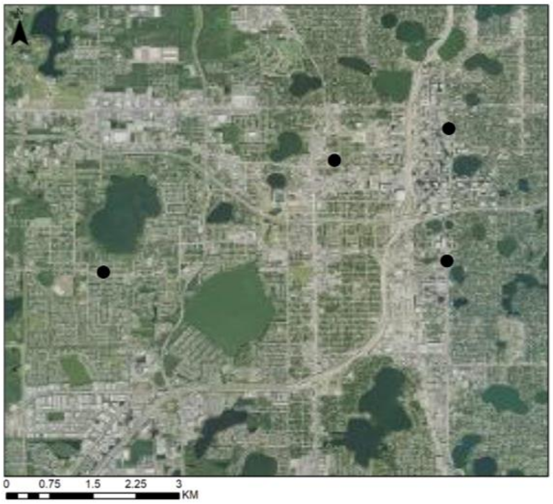

Tampa, FL

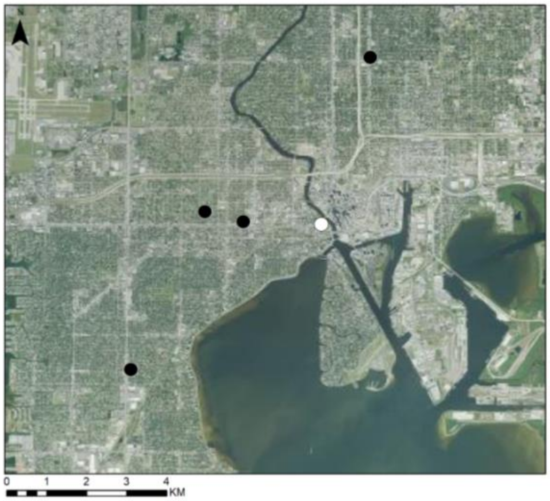

Sarasota, FL

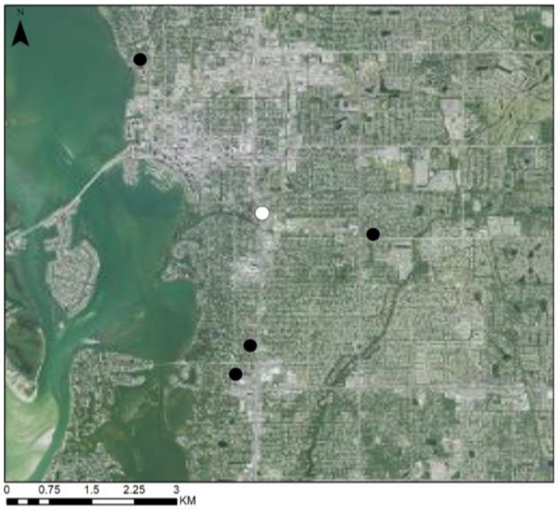

Fort Myers, FL

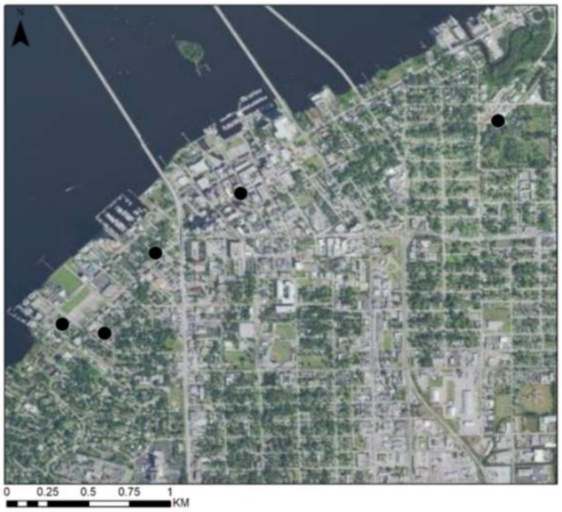

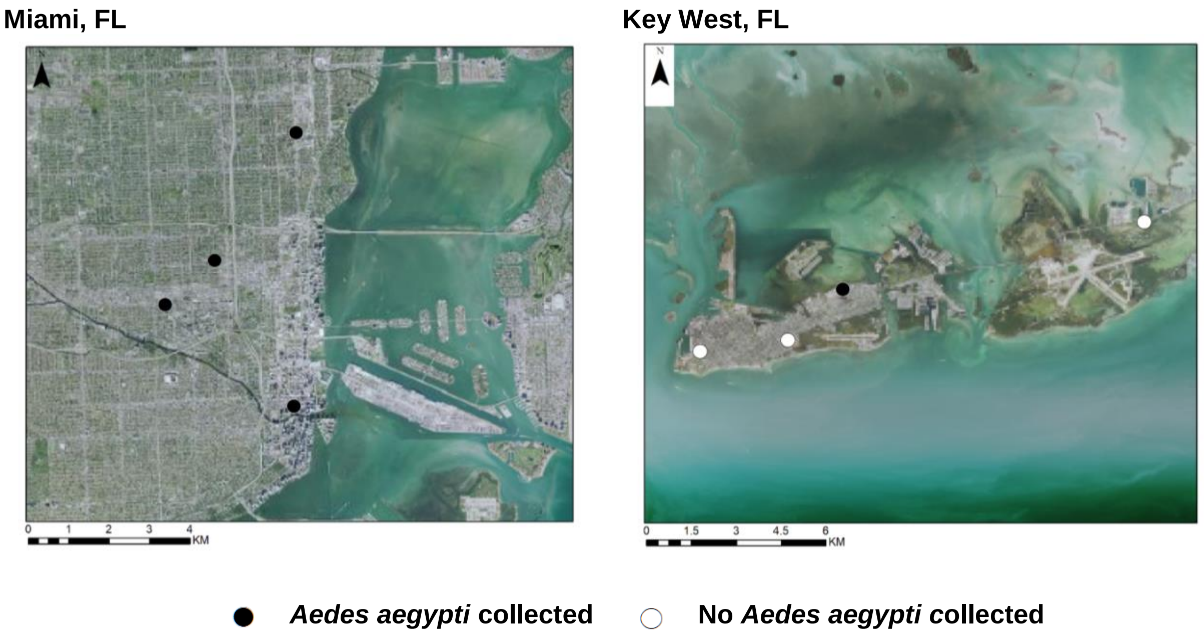

Additional file 1: Figure S1. ***Aedes aegypti*** **collection locations within cities.** Multiple ovitraps and/or larval/adult sampling were conducted at each collection location. Black circles indicate *Aedes aegypti* specimens were collected at that location and were used in this study. White circles indicate no *Aedes aegypti* specimens were recovered from that location. Points are overlaid 2017 NAIP imagery, reprinted from the USGS (https://catalog.data.gov/dataset/usgs-naipplus-overlay-map-service-from-the-national-map), public domain, original copyright 2017.
