# Supplementary material for: Patterns of genetic divergence among populations of Aedes aegypti L. (Diptera: Culicidae) in the southeastern USA
Source: Parasit Vectors. 2019 Oct 30;12:511. doi: 10.1186/s13071-019-3769-0 (PMC6822358; doi:10.1186/s13071-019-3769-0)
Supplement: Supplementary file 2 — Additional file 2: Table S1. Microsatellite loci used in study. Loci pairs are based on non-overlapping size ranges. Size range is derived from a literature review [30–32, 55]. 470AG1 was excluded due to significant deviations from Hardy-Weinberg. [file 13071_2019_3769_MOESM2_ESM.docx]

**Additional file 2: Table S1. Microsatellite loci used in study.** Loci pairs are based on non-overlapping size ranges. Size range was derived from a literature review [30-32, 55]. 470AG1 was excluded due to significant deviations from Hardy Weinberg.

| Locus | Motif | Size Range | Source |
| --- | --- | --- | --- |
| A1 | Tri- | 149-174 | Brown [31] |
| A9 | Tri- | 181-197 | Brown [31] |
| B2 | Tri- | 95-113 | Brown [31] |
| B3 | Tri- | 148-178 | Brown [31] |
| CT2 | Di- | 182-196 | Slotman [32] |
| AC1 | Di- | 195-211 | Slotman [32] |
| AC2 | Di- | 182-192 | Slotman [32] |
| AC5 | Di- | 145-169 | Slotman [32] |
| 1132CT1 | Di- | 151-203 | Lovin [30] |
| 470AG1 | Di- | 227-255 | Lovin [30] |
