# Supplementary material for: Patterns of genetic divergence among populations of Aedes aegypti L. (Diptera: Culicidae) in the southeastern USA
Source: Parasit Vectors. 2019 Oct 30;12:511. doi: 10.1186/s13071-019-3769-0 (PMC6822358; doi:10.1186/s13071-019-3769-0)
Supplement: Supplementary file 4 — Additional file 4: Table S2. Population-by-locus genetic diversity of microsatellite markers in eight Aedes aegypti populations. N is the number of individuals, NA is number of alleles, HO is observed heterozygosity, HE is expected heterozygosity, and P is P-value of an exact test. Note that * indicates P < 0.05 with corrections for multiple comparisons; d is heterozygote deficiency and e is heterozygote excess. [file 13071_2019_3769_MOESM4_ESM.docx]

**Additional file 4: Table S2. Population-by-locus genetic diversity of microsatellite markers in eight *Aedes aegypti* populations.** *N* is the number of individuals, *N_A_* is number of alleles, *H_O_* is observed heterozygosity, *H_E_* is expected heterozygosity, and *P* is p-value of an exact test. Note that * indicates *P* < 0.05 with corrections for multiple comparisons; *d* is heterozygote deficiency, and *e* is heterozygote excess.

| **Population** | **Locus** | ***N*** | ***N_A_*** | ***H_O_*** | ***H_E_*** | ***P*** |
| --- | --- | --- | --- | --- | --- | --- |
| Columbus, GA | A1 | 24 | 4 | 0.375 | 0.674 | 0.014 |
|  | A9 | 22 | 4 | 0.318 | 0.445 | 0.076 |
|  | B2 | 21 | 5 | 0.667 | 0.522 | 0.945 |
|  | B3 | 22 | 3 | 0.364 | 0.607 | < 0.001* |
|  | CT2 | 22 | 2 | 0.227 | 0.499 | 0.012 |
|  | AC1 | 23 | 5 | 0.783 | 0.726 | 0.244 |
|  | AC2 | 24 | 3 | 0.625 | 0.582 | 0.021 |
|  | AC5 | 23 | 6 | 0.826 | 0.721 | 0.422 |
|  | 1132CT1 | 22 | 6 | 0.455 | 0.455 | 0.515 |
| Daytona, FL | A1 | 29 | 5 | 0.621 | 0.679 | 0.002* |
|  | A9 | 26 | 3 | 0.308 | 0.630 | 0.003*^,^*^d^* |
|  | B2 | 28 | 6 | 0.786 | 0.644 | 0.546 |
|  | B3 | 28 | 4 | 0.393 | 0.476 | 0.189 |
|  | CT2 | 27 | 2 | 0.481 | 0.497 | 1.000 |
|  | AC1 | 29 | 5 | 0.483 | 0.632 | 0.017 |
|  | AC2 | 29 | 4 | 0.276 | 0.323 | 0.077 |
|  | AC5 | 29 | 7 | 0.690 | 0.792 | 0.061 |
|  | 1132CT1 | 28 | 7 | 0.607 | 0.584 | 0.183 |
| Orlando, FL | A1 | 28 | 5 | 0.607 | 0.692 | 0.023 |
|  | A9 | 28 | 3 | 0.536 | 0.448 | 1.000 |
|  | B2 | 22 | 7 | 0.773 | 0.662 | 0.698 |
|  | B3 | 28 | 3 | 0.571 | 0.641 | 0.670 |
|  | CT2 | 28 | 2 | 0.393 | 0.392 | 1.000 |
|  | AC1 | 28 | 4 | 0.821 | 0.669 | 0.003* |
|  | AC2 | 28 | 4 | 0.429 | 0.362 | 1.000 |
|  | AC5 | 28 | 7 | 0.643 | 0.807 | 0.030 |
|  | 1132CT1 | 28 | 11 | 0.643 | 0.801 | < 0.001* |
| Tampa, FL | A1 | 28 | 5 | 0.714 | 0.723 | 0.051 |
|  | A9 | 28 | 4 | 0.536 | 0.677 | 0.234 |
|  | B2 | 25 | 7 | 0.680 | 0.610 | 0.177 |
|  | B3 | 27 | 3 | 0.370 | 0.615 | 0.004*^,^ *^d^* |
|  | CT2 | 27 | 3 | 0.519 | 0.479 | 1.000 |
|  | AC1 | 28 | 5 | 0.464 | 0.555 | 0.034 |
|  | AC2 | 28 | 4 | 0.250 | 0.391 | 0.008 |
|  | AC5 | 28 | 7 | 0.571 | 0.813 | < 0.001*^,^ *^d^* |
|  | 1132CT1 | 27 | 12 | 0.519 | 0.785 | < 0.001*^,^ *^d^* |
| Sarasota, FL | A1 | 29 | 4 | 0.759 | 0.613 | 0.004* |
|  | A9 | 29 | 4 | 0.345 | 0.599 | 0.008 |
|  | B2 | 24 | 7 | 0.875 | 0.691 | 0.209 |
|  | B3 | 26 | 4 | 0.615 | 0.668 | 0.833 |
|  | CT2 | 25 | 2 | 0.280 | 0.449 | 0.075 |
|  | AC1 | 29 | 5 | 0.724 | 0.696 | 0.019 |
|  | AC2 | 28 | 4 | 0.571 | 0.663 | 0.001* |
|  | AC5 | 29 | 7 | 0.793 | 0.786 | 0.336 |
|  | 1132CT1 | 27 | 7 | 0.444 | 0.663 | < 0.001* |

| **Population** | **Locus** | ***N*** | ***N_A_*** | ***H_O_*** | ***H_E_*** | ***P*** |
| --- | --- | --- | --- | --- | --- | --- |
| Fort Myers, FL | A1 | 29 | 4 | 0.379 | 0.588 | < 0.001* |
|  | A9 | 28 | 3 | 0.500 | 0.645 | 0.066 |
|  | B2 | 26 | 6 | 0.885 | 0.737 | 0.005* |
|  | B3 | 26 | 3 | 0.846 | 0.607 | 0.094 |
|  | CT2 | 27 | 2 | 0.259 | 0.366 | 0.135 |
|  | AC1 | 29 | 5 | 0.690 | 0.740 | < 0.001* |
|  | AC2 | 29 | 4 | 0.586 | 0.539 | 1.000 |
|  | AC5 | 29 | 7 | 0.655 | 0.754 | 0.025 |
|  | 1132CT1 | 27 | 8 | 0.556 | 0.697 | 0.057 |
| Miami, FL | A1 | 29 | 4 | 0.586 | 0.669 | 0.018 |
|  | A9 | 29 | 3 | 0.655 | 0.651 | 0.764 |
|  | B2 | 28 | 7 | 0.821 | 0.742 | 0.095 |
|  | B3 | 28 | 4 | 0.607 | 0.621 | 0.200 |
|  | CT2 | 27 | 3 | 0.444 | 0.510 | 0.691 |
|  | AC1 | 29 | 4 | 0.483 | 0.590 | 0.147 |
|  | AC2 | 29 | 2 | 0.552 | 0.400 | 0.069 |
|  | AC5 | 28 | 8 | 0.500 | 0.719 | < 0.001*^,^ *^d^* |
|  | 1132CT1 | 27 | 10 | 0.593 | 0.619 | 0.226 |
| Key West, FL | A1 | 21 | 3 | 0.571 | 0.571 | 1.000 |
|  | A9 | 21 | 4 | 0.429 | 0.455 | 0.115 |
|  | B2 | 21 | 6 | 1.000 | 0.736 | 0.001*^,^ *^e^* |
|  | B3 | 21 | 3 | 0.619 | 0.580 | 0.265 |
|  | CT2 | 21 | 2 | 0.619 | 0.495 | 0.394 |
|  | AC1 | 21 | 4 | 0.905 | 0.698 | 0.105 |
|  | AC2 | 21 | 3 | 0.429 | 0.359 | 1.000 |
|  | AC5 | 21 | 5 | 0.667 | 0.763 | 0.452 |
|  | 1132CT1 | 21 | 8 | 0.952 | 0.822 | 0.113 |
