# Supplementary material for: Patterns of genetic divergence among populations of Aedes aegypti L. (Diptera: Culicidae) in the southeastern USA
Source: Parasit Vectors. 2019 Oct 30;12:511. doi: 10.1186/s13071-019-3769-0 (PMC6822358; doi:10.1186/s13071-019-3769-0)
Supplement: Supplementary file 5 — Additional file 5: Figure S2. Signature of isolation by distance for both Euclidean distance and driving distance. Scatterplots of Euclidean distance (a) and driving distances (b) by pairwise linear FST with fitted linear regressions superimposed. [file 13071_2019_3769_MOESM5_ESM.docx]

**a**


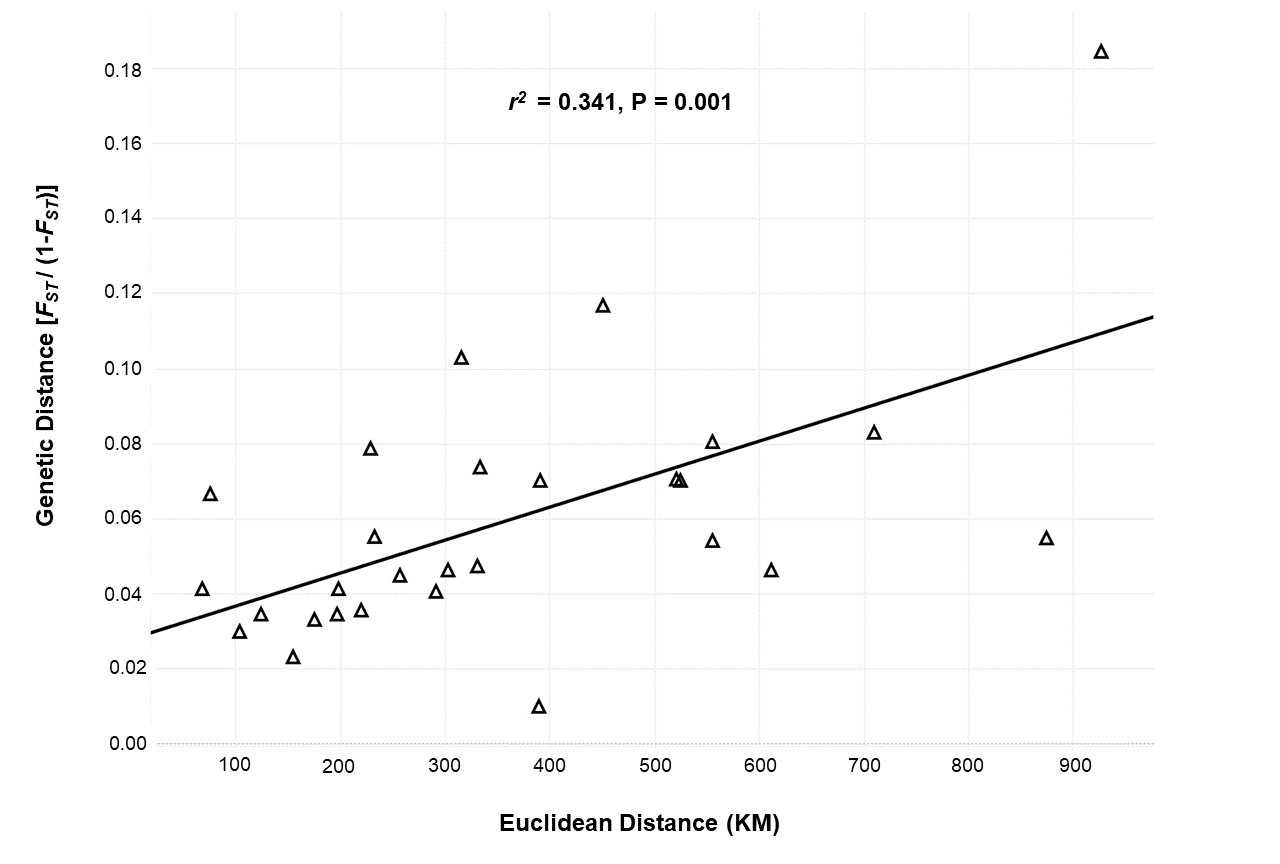


**b**


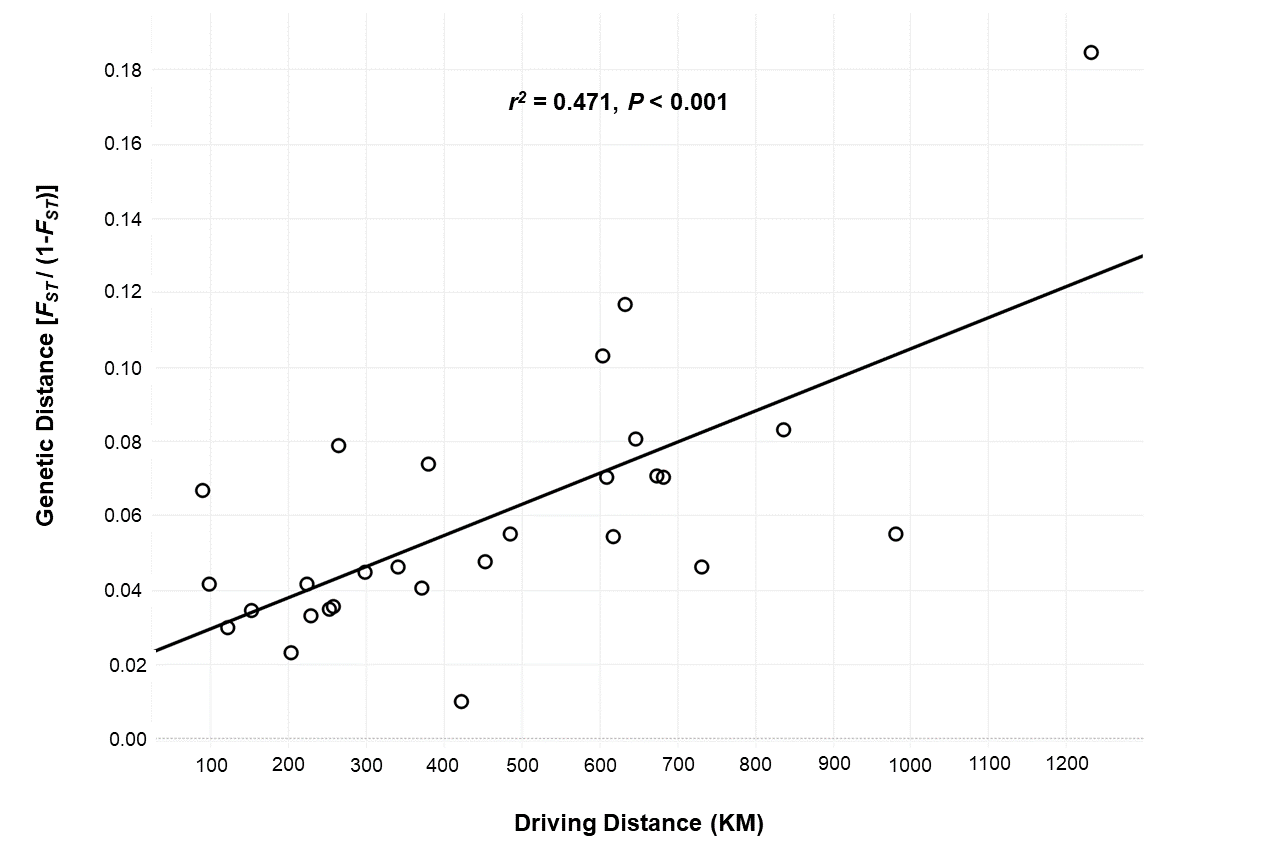


**Additional file 5: Figure S2. Signature of isolation by distance for both Euclidean distance and driving distance.** Scatterplots of Euclidean distance (A) and driving distances (B) by pairwise linear F_ST_ with fitted linear regressions superimposed.
