# Supplementary material for: Patterns of genetic divergence among populations of Aedes aegypti L. (Diptera: Culicidae) in the southeastern USA
Source: Parasit Vectors. 2019 Oct 30;12:511. doi: 10.1186/s13071-019-3769-0 (PMC6822358; doi:10.1186/s13071-019-3769-0)

**Additional file 6: Figure S3. Delta K analysis of the true number of clusters following the Evanno method.**


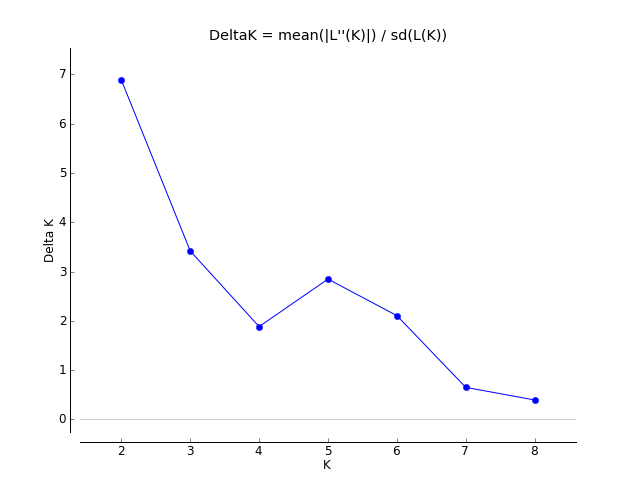

Supplement: Supplementary file 6 — Additional file 6: Figure S3. Delta K analysis of the true number of clusters following the Evanno method. [file 13071_2019_3769_MOESM6_ESM.docx]
