# Supplementary material for: Patterns of genetic divergence among populations of Aedes aegypti L. (Diptera: Culicidae) in the southeastern USA
Source: Parasit Vectors. 2019 Oct 30;12:511. doi: 10.1186/s13071-019-3769-0 (PMC6822358; doi:10.1186/s13071-019-3769-0)
Supplement: Supplementary file 7 — Additional file 7: Figure S4. Discriminant analysis of principal components (DAPC). Analysis with 40 principal components and a cross-validation of 100 iterations (mean success = 0.509, RMSE = 0.502). [file 13071_2019_3769_MOESM7_ESM.docx]

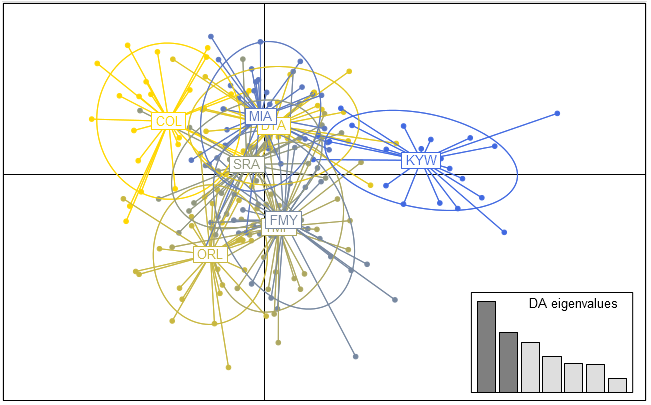


**Additional file 7: Figure S4. Discriminant analysis of principal components (DAPC).** Analysis with 40 principal components and a cross-validation of 100 iterations (mean success = 0.509, RMSE = 0.502).
